# Flap endonuclease 1 Facilitated Hepatocellular Carcinoma Progression by Enhancing USP7/MDM2-mediated P53 Inactivation

**DOI:** 10.7150/ijbs.68179

**Published:** 2022-01-01

**Authors:** Saiyan Bian, Wenkai Ni, Mengqi Zhu, Xue Zhang, Yuwei Qiang, Jianping Zhang, Zhiyu Ni, Yiping Shen, Shi Qiu, Qianqian Song, Mingbing Xiao, Wenjie Zheng

**Affiliations:** 1 Research Center of Clinical Medicine, Affiliated Hospital of Nantong University, Nantong 226001, P.R. China.; 2 School of Medicine, Nantong University, Nantong 226001, P.R. China.; 3 Department of Oncology, Affiliated Hospital of Nantong University, Nantong 226001, P.R. China.; 4 Department of Radiology, Wake Forest School of Medicine, Winston-Salem 27157, USA.

**Keywords:** Flap endonuclease 1, hepatocellular carcinoma, P53, mouse double minute 2, ubiquitin-specific protease 7, molecular target

## Abstract

Overexpression of Flap endonuclease 1 (FEN1) has been previously implicated in hepatocellular carcinoma (HCC), while its expression features and mechanisms remain unclear. In the current study, differential expression genes (DEGs) were screened in HCC tissues and normal liver tissues in 4 Gene Expression Omnibus (GEO) datasets. FEN1, one of the hub co-overexpressed genes, was further determined overexpressed in HCC tissues in TCGA, local HCC cohorts, and hepatocarcinogenesis model. In addition, high expression of FEN1 indicated poor prognosis of HCC patients. Loss-of-function and gain-of-function assays demonstrated that FEN1 enhanced the proliferation, cell cycle phage transition, migration/ invasion, therapy resistance, xenograft growth, and epithelial-mesenchymal transition (EMT) process of HCC cells. Mechanically, FEN1 could inactivate P53 signaling by preventing the ubiquitination and degradation of mouse double minute 2 (MDM2) via recruiting ubiquitin-specific protease 7 (USP7). Interfering USP7 with P22077 significantly reversed the malignant phenotypes activated by FEN1. In conclusion, this study suggests FEN1 as a robust prognostic biomarker and potential target for HCC.

## Introduction

Hepatocellular carcinoma (HCC), accounting for 80 % of the liver tumors, ranks the sixth most common malignancy with the leading mortality rate worldwide 1. The high incidence of HCC is attributed to various risks factors, including hepatitis B or hepatitis C virus infection, aflatoxin, diabetes, obesity, alcohol abuse, and even smoking 2, 3. In recent years, remarkable progressions have been observed in the surveillance, diagnosis, and treatment of HCC. Surgery resection is common choice of HCC patients at early stages. However, patients at advanced stages still suffer the poor prognosis due to high risks of recurrence and metastasis. Although some molecular targeted agents like sorafenib (first-line), regorafenib (second-line), and cabozantinib have shown certain benefits to some degree, low chemo-response and the liver function requirements limit the application in advanced HCC 4. Thus, it is critical to determine the mechanism of malignant phenotypes and explore therapeutic targets for HCC.

Flap endonuclease 1 (FEN1), known as a flap structure-specific and multifunctional endonuclease, is a member of the RAD2/XPG superfamily 5. FEN1 handles the removal of 5'-flap structures formation in Okazaki fragment maturation and long-patch base excision repair by cleaving within the apurinic/apyrimidinic (AP) site-terminated flap 6. Additionally, FEN1 possesses 5'-3' exonuclease activities and gap endonuclease (GEN) activities, thereby promoting fragmentation of the DNA secondary structures, telomere maintenance, and rDNA replication 7. As a consequence, dysregulated FEN1 expression leads to genetic diseases, including Werner syndrome, Vitelliform macular dystrophy, and Bloom syndrome (BLM) 5, 8, 9.

During physiological and pathological processes, protein-protein interactions are observed between FEN1 and various DNA replication and repairing-related proteins. Increasing evidence has indicated the pro-tumorigenesis functions of various cancer types 10. A previous meta-analysis demonstrated that FEN1 polymorphisms at rs174538/ rs4246215 reduced the risks of various cancer types 11, 12. FEN1 overexpression indicated poor survival and facilitated malignant phenotypes and chemoresistance of non-small-cell lung cancer (NSCLC)^13^. Exogenous FEN1 promoted proliferation and tumor growth of breast cancer cells, which was abrogated by its inhibitor SC13 by retarding DNA replication *in vitro* and* in vivo*^14^. Additionally, down-regulating FEN1 of breast cancer cells by letrozole enhanced the cisplatin sensitivity via ERK/Elk-1 signaling 15.

Actually, a recent study correlated FEN1 with HBV covalently closed circular DNA (cccDNA), which served as the template for all HBV transcripts 16. FEN1 protein binds and cleaves the 5'-flap structure of HBV relaxed circular DNA (rcDNA), thereby facilitating cccDNA conversion. However, the expression features, underlying functions and mechanism in HCC remain unknown. In the current study, FEN1, a prognosis-related hub gene validated in bioinformatic/ local HCC cohorts and hepatocarcinogenesis model, was further investigated to discover its molecular mechanisms in HCC progression.

## Materials and methods

### Information of local HCC cohort

163 pairs of HCC specimens and corresponding para-cancerous tissues were obtained from HCC patients received hepatectomy in Affiliated Hospital of Nantong University (Nantong, Jiangsu, China) from October 2010 to January 2014. All HCC Samples were confirmed histologically. The study was designed and performed according to the Helsinki Declaration and approved by the ethic committee of Affiliated Hospital of Nantong University.

### Cell culture and transfection

SK-HEP1(catalog number, #ZQ0030), HepG2 (#ZQ0022), Hep3B (#ZQ0024), Huh7 (#ZQ0025), SMMC-7721 (#ZQ0029), HCCLM3 (#ZQ0023), and LO2 (#ZQ0031) were purchased from Zhong Qiao Xin Zhou Biotechnology (Shanghai, China). The cells were cultured in Dulbecco's modified Eagle's medium (DMEM, Hyclone, CA, USA) or RIPM1640 medium supplemented with fetal bovine serum (FBS; Gibco, CA, USA) and penicillin/streptomycin at 37 °C under the condition of 5% CO2. Human FEN1 cDNA was amplified by PCR and cloned into the pTSB02-GFP-PURO vector constructed by Transheep (Shanghai, China). siRNA Smartpool targeting FEN1 was designed and constructed by Dharmocon (NY, USA). Plasmids or siRNAs were transfected with Lipofectamine 3000 reagent (Invitrogen, CA, USA) according to the manufacturer's instructions. Cells transfected with pTSB02-GFP-PURO plasmids were selected with puromycin (5 μg/mL, Sigma-Aldrich, CA, USA) for 30 days to obtain stably transfected cells.

### Data processing and bioinformatic analyses

Microarray data of GSE14520-GPL3921 (220 liver non-tumor tissues and 225 HCC tissues), GSE14520-GPL571 (21 liver non-tumor tissues and 22 HCC tissues), GSE25097 (300 HCC tissues, 40 liver cirrhotic tissues and 6 normal livers), GSE121248 (37 adjacent tissues and 70 HCC tissues), GSE50579 (24 HCC tissues), GSE6764 (35 HCC tissues, 30 liver cirrhotic and dysplasia tissues) , and GSE17548 (17 HCC tissues, 20 liver cirrhotic tissues) was obtained from Gene Expression Omnibus (GEO) database (http://www.ncbi.nlm.nih.gov/geo/). The RNA-sequencing data of LIHC was downloaded from The Cancer Genome Atlas Liver Hepatocellular Carcinoma (TCGA-LIHC; https://portal.gdc.cancer.gov). Profiles as Fragments Per Kilobase per Million (FPKM) data was performed Log2 transformation into transcripts per million reads (TPM). R packages “survival” and “survminer” were used to perform Univariate and Multivariate Cox regression analyses and calculate Kaplan-Meier analyses. Nomograms were established with R packages “survival” and “rms”. R package “timeROC” was performed to plot time-dependent ROC. The DEGs between HCC and normal tissues or FEN1-high and FEN1-low groups were identified by conducting R package “deseq2” with the standard of False discovery rate |logFC| > 1 and P<0.05. P values were adjusted by Benjamini-Hochberg (BH) method 17. R package “clusterProfiler” was performed for functional or pathway enrichment analysis of DEGs with the standard of False discovery rate (FDR) < 0.25 and P.adjust < 0.05. The bioinformatic analyses were conducted based on R version 3.6.3.

### Cell viability and colony formation assays

Cell proliferation and drug sensitivity were detected by Cell Counting Kit-8 assay (CCK-8; Dojindo Laboratories, Kumamoto, Japan) according to the manufacturer's protocols. Cells with different treatment were cultured in 96-well plates at a density of 1 × 10^3^ cells per well. CCK-8 solution was administrated at indicated time points. After incubated at 37 °C for 2 h, OD450 values of each well were detected by a microplate reader (BioTeK, CA, USA). For colony formation assays, cells were cultured in 6-well plates at a density of 500/well. After incubation for 14 days, the samples were fixed in 4% paraformaldehyde (PFA) for 30 min and stained with crystal violet solution.

### Flow cytometry

The distribution of cell cycle and cell apoptosis was analyzed by flow cytometry according to the manufacturer's protocols. For cell cycle detection, cells were harvested with trypsin, followed by resuspending in PBS at the concentration of 1×10^5^/100 μL. Then cells were stained with PI for 30 min on ice. Following washing with PBS, samples were detected by using a BD FACS Calibur flow cytometer (USA). The cell cycle distribution was analyzed by ModFit software. For apoptosis detection, cells were washed and resuspended in pre-cold PBS. Then the samples were incubated in Annexin V-647 and PI solution and protected from light. After exposure of 15 min, the apoptosis was detected by the flow cytometer.

### Migration, invasion, and wound-healing assays

Transwell assays were performed to test the effects of FEN1 knockdown/ overexpression on migration and invasion. The general protocol was performed as previously described 18. In brief, 4 × 10^4^ cells were pre-suspended in serum-free medium and plated in the transwell inserts (Corning, CA, USA) for migration assay, while 1 × 10^5^ cells suspended in 200 μl serum-free medium were seeded in the Matrigel-precoated Transwell inserts (Corning Life Sciences, MA, USA) for invasion assay. The complete culture medium was placed in the lower chamber. Following incubation for 24 h, the chambers were fixed by PFA and stained with crystal violet. After carefully rinsed with pure water, the migration or invasion cells were counted in 3 random visual fields. For wound-healing assay, cells were maintained in serum-free medium in 6-well plates to avoid the effects of proliferation. The line wounds were scratched on the confluent cell monolayers by using a 200 μl pipette tip. The relative distance (RD) of each group was calculated with the equation: RD= (D_0 h_ -D_24 h_) ⁄ D_0 h_.

### 3D sphere growth and 3D invasion assay

The 3D tumor spheroid growth assay was conducted as previously described 18. In brief, 1000 single-cell suspended cells in 50 µL were cultured in an ultra-low attachment 96-well plate (Corning, New York). Following adding 50 µL media in 24 h, the spheroids were measured at the time point of 24h, 72h, and 168h, respectively. The 3D invasion assay was performed according to a previous study 19. 500 cells in culture medium were seeded in an ultra-low attachment 96-well plate to form tumor spheroids. Then, the spheroids were collected and embedded into 3D matrix mixture (Matrigel and type I collagen in a ratio of 1:1). The mixtures were placed in 24-well plate and cultured for 72h at 37 ℃. The 3D invasion was observed under a microscope.

### RT-qPCR

Total RNA was isolated by TRIzol reagent (Thermo, USA). The cDNA was synthesized by performing a ReverTra Ace qPCR RT Kit (Toyobo, Japan). RT-qPCR was conducted by using The Fast SYBR Green Master Mix (Applied Biosystems Inc., MA, United States) according to the manufacturer's instructions. The condition of PCR was set as follows: 30 s polymerase activation at 95°C and 40 cycles at 95°C for 5 s, followed by 60°C for 30 s. GAPDH was employed as the control. The relative expression of target gene was calculated by the 2^-ΔΔCT^ method. The sequences of the primers were listed in **Table S1**.

### Immunohistochemistry

Formaldehyde-fixed and paraffin- embedded tissues were dewaxed and dehydrated by xylene and serial diluted ethanol, respectively. Following treated with hydrogen peroxide and blocked with BSA, the slides were exposed to primary antibodies of FEN1 (1:200, Abcam, USA, USA), P53 (1:200, Proteintech, MA, USA), MDM2 (1:200, Proteintech, MA, USA), USP7 (1:200, MA, Proteintech) and Ki67 (1:200, MA, Proteintech) at 4 °C for 12h. After rinsed with TBST, the sections were incubated in streptavidin-biotin complex and visualized by using 3,3′-diaminobenzidine (DAB)/ hematoxylin. The histological staining of the slides was independently evaluated by two experienced pathologists. The score of immunohistochemistry was based on the staining intensity and the positive cells in six random microscopic fields.

### Immunoprecipitation and protein stability assay

The ubiquitin immunoprecipitation was conducted as previously described 18. The cell lysates were extracted by RIPA on ice for 30 min, followed by incubation in primary MDM2 antibody (Santa Cruz Biotechnology, CA, USA) at 4 °C for 12h. Subsequently, we placed the samples in anti-rabbit Ig-IP beads (Rockland Immunochemicals, USA) with continuous spun down at 4 °C for 2.5 h. Then the proteins isolated from the beads were subjected to immunoblotting. For protein stability assay, cells were administrated with CHX (100 μg/ml, Sigma, CA, USA) and collected at different time points, thereby conducting western blotting to detect the protein degradation.

### Rat hepatocarcinogenesis model

The rat hepatocarcinogenesis model was developed as previously described 20. 4-6 week-old Sprague-Dawley rats were provided by the Animal Research Center of Nantong University. The rats were randomized into control group (n = 10) and the hepatocarcinogenesis group (n = 30), which were fed with normal diet and 2-acetylaminofluorene (2-AAF, Sigma, CA, USA), respectively. After histologically and pathologically confirmed, the rats in hepatocarcinogenesis group were stratified into degeneration subgroup (n = 6), precancerous subgroup (n = 6), and HCC subgroup (n = 6). The resected livers from the groups above were collected for further validations. The procedures were approved by the Animal Care and Use Committee of Nantong University.

### Xenograft model

6-8 week-old Balb/c mice were obtained from the Animal Research Center of Nantong University. To test the effects of FEN1 on tumor growth* in vivo*, FEN1-overexpressed or negative control (NC) HepG2 cells (1 × 10^6^) were randomly and subcutaneously injected into the nude mice. The volume of xenograft was calculated as the equation: xenograft volume = 1/2 (length × width^2^). Mice were sacrificed after 4 weeks, and the xenografts were resected and weighed. All procedures were approved by the Animal Care and Use Committee of Nantong University.

### Western blotting

Protein was extracted by RIPA containing the protease inhibitor. Then, the proteins were separated on a sodium dodecyl sulfate polyacrylamide gel electrophoresis (SDS-PAGE) and transferred onto polyvinylidene difluoride (PVDF) membranes. Following blocked in 5% BSA for 2 hours, the membranes were incubated in primary antibodies at 4 °C overnight. After that, the membranes were rinsed TBS and probed with HPR-conjugated secondary antibodies at room temperature for 2 hours. Subsequently, the bands were detcted by enhanced chemiluminescence (ECL) kit (Millipore, CA, USA).

### Statistical analysis

IBM SPSS19.0, GraphPad Prism 7.0 (CA, USA) and R software were used for statistical analysis. All data were derived from at least three repeated experiments. The* χ*^2^ test and Student's t-test were performed to evaluate the differences between two groups, and the ANOVA was used for comparisons of multiple groups. P value less than 0.05 was considered statistically significant.

## Results

### FEN1 was a hub gene of DEGs between HCC tissues and normal liver tissues

**Figure 1:**
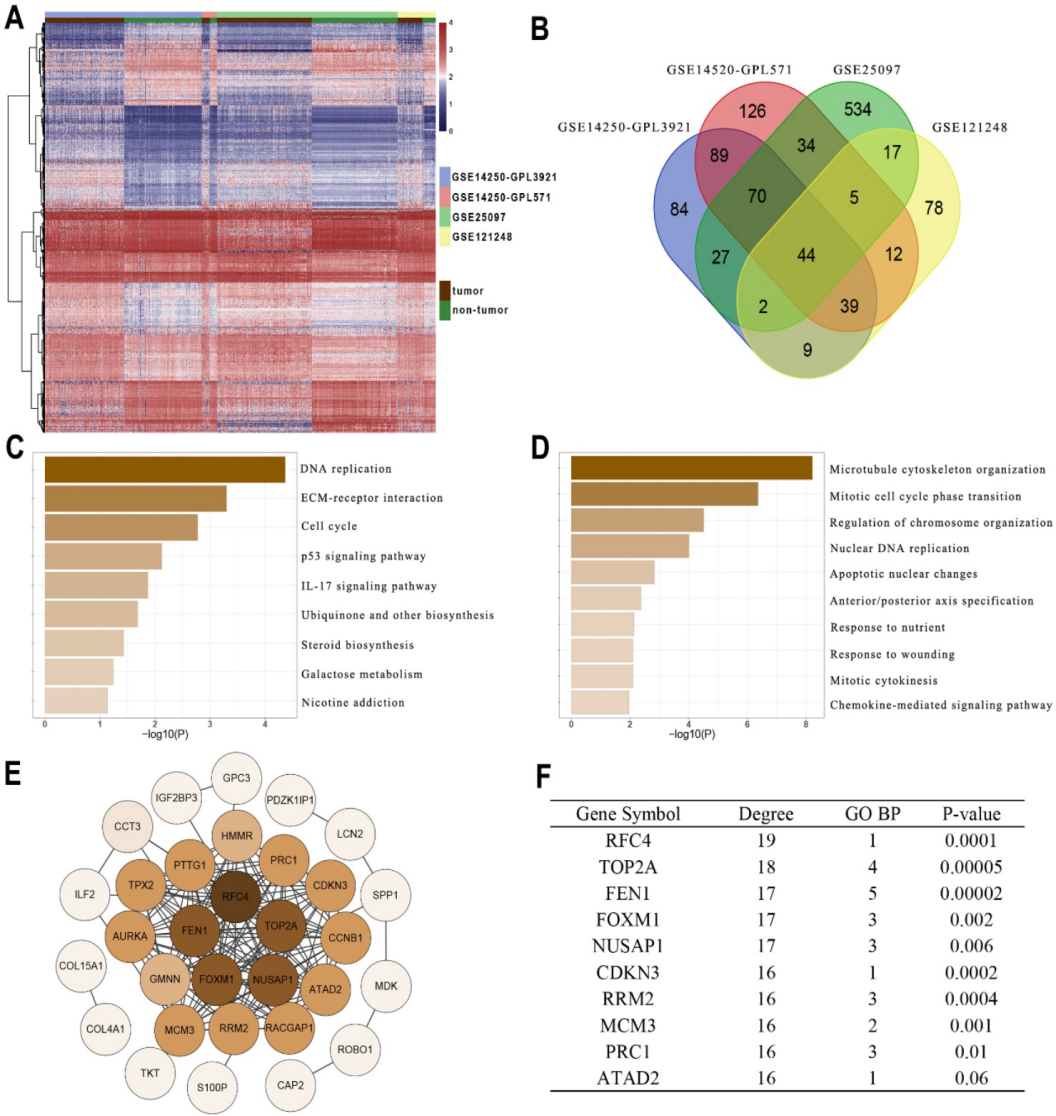
** Identifying FEN1 as a hub gene of HCC. (A)** Heatmap showed the upregulated genes between HCC and normal liver samples in multiple GEO datasets. DEGs were screened with the standard log2(FC)>1 & P.adj<0.05. **(B)** Venn plot of the co-overexpressed genes in the four datasets. **(C)** KEGG enrichment analysis of the genes. **(D)** GO_BP enrichment analysis of the genes. **(E)** Protein-Protein interaction analysis of the genes was performed by STRING. **(F)** Node degree, frequency in TOP5 enriched pathways, and Log-rank test P-values of the hub genes of HCC.

Gene profiles were extracted from four GEO datasets (GSE14250-GPL3921, GSE14520-GPL571, GSE25097, and GSE121248) and presented in the heatmap **(Figure 1A)**. A total of 44 DEGs were co-overexpressed in the 4 datasets with a criterion of logFC > 1 and Adj.P < 0.05 **(Figure 1B)**. Furthermore, the enrichment analysis indicated that the DEGs were mainly involved in the pathways including DNA replication, cell cycle, P53 signaling, IL-17 signaling, and ECM interactions **(Figure 1C)**. In addition, the DEGs might also be correlated with processes including chromosome organization, apoptosis, cytokines and chemokines** (Figure 1D)**. Then String was performed to observe the interactions among these genes **(Figure 1E)**. According to **Figure 1F**, FEN1 had 17 node degrees of the protein-protein interaction (ranking the 3^rd^ in the DEGs), 5 enrichments in TOP5 GO pathways (ranking 1^st^ in the DEGs), and superior prognostic significance for HCC (0.00002, ranking 2^nd^ in DEGs). Based on these analyses, FEN1, a potential hub gene of HCC, was screened for further validations.

### The expression features of FEN1 in hepatocarcinogenesis and HCC progression

**Figure 2:**
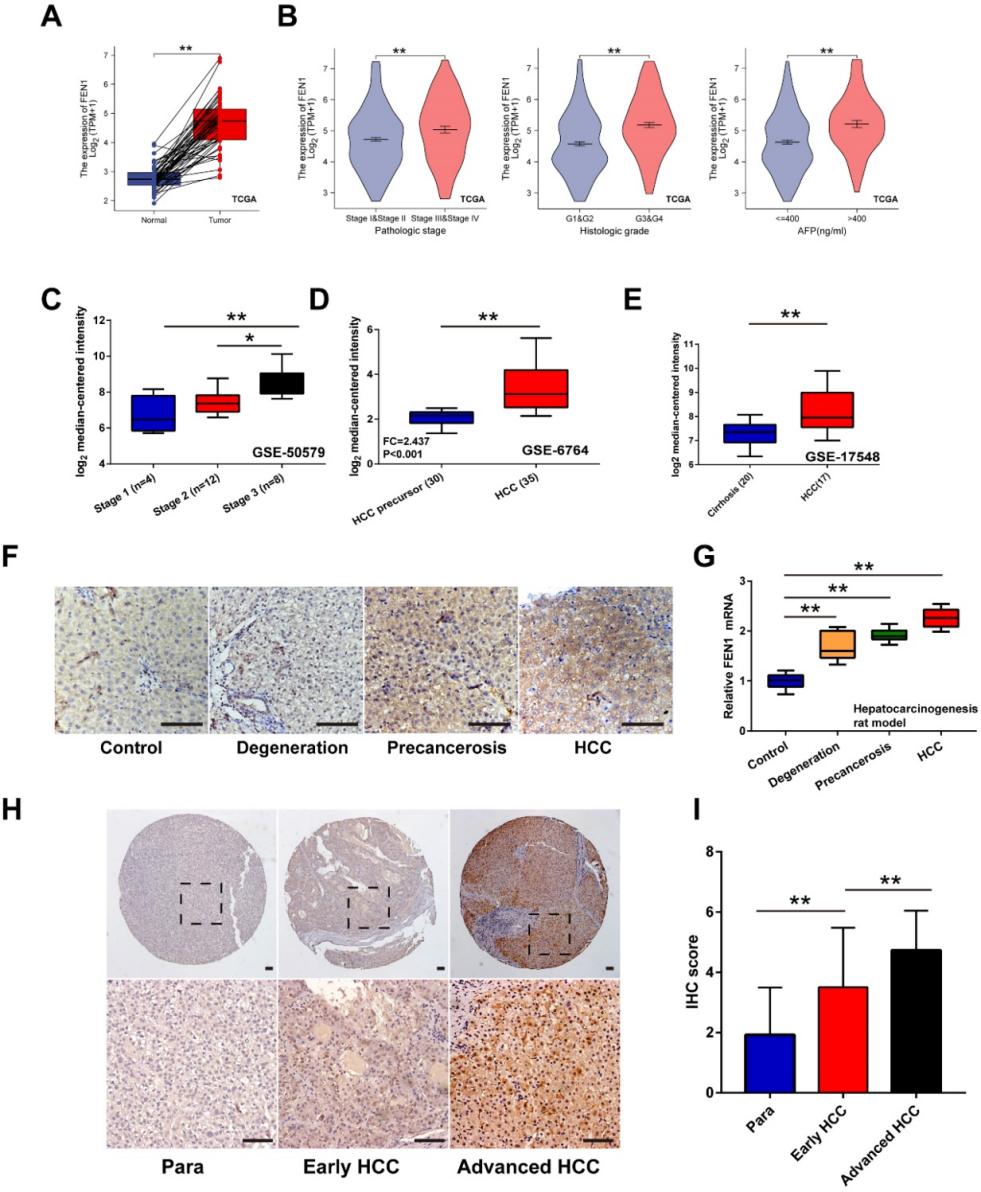
** The expression features of FEN1 in HCC. (A)** FEN1 expression in 50 pairs of HCC and matched normal tissues in TCGA LIHC dataset.** (B)** The FEN1 expression in HCC patients with different pathologic stages, histologic grades, and AFP levels in TCGA LIHC dataset. **(C)** The FEN1 expression in HCC patients with different stages in GSE50579. **(D)** The FEN1 expression in tumor precursor and HCC tissues in GSE6764. HCC precursor: liver cirrhosis and dysplasia. **(E)** The FEN1 expression in cirrhotic tissues and HCC tissues in GSE17548. **(F)** The immunohistochemistry of the FEN1 in the rat hepatocarcinogenesis model.** (G)** mRNA levels of FEN1 in the rat hepatocarcinogenesis model detected by RT-qPCR.** (H)** Representative immunohistochemical staining of the FEN1 in a local cohort including 183 self-matched HCC and para-cancerous tissues. **(I)** The semi-quantitative score of immunohistochemical staining of IHC. **, P<0.01.

Firstly, overexpression of FEN1 was determined in HCC tissues of 50 pairs matched tumor/ adjacent cases **(Figure 2A)**. Compared with normal tissues, higher expression of FEN1 was also observed in HCC tissues according to external 9 GEO datasets and ICGC dataset (**Figure S1**). Focusing on the subgroups of TCGA datasets, FEN1 expression was elevated in HCC patients with advanced pathologic stages/ histologic grades, and high AFP levels **(Figure 2B)**. In consistence, omics data in GSE50579 also indicated that patients with advanced TNM stages had higher FEN1 expression **(Figure 2C)**. Interestingly, FEN1 might be involved in the hepatocarcinogenesis based on the investigation in two external GEO datasets **(Figure 2D and E).** Then, we evaluated the FEN1 alteration in the pre-established rat hepatocarcinogenesis model. Dynamically increased expression of FEN1 was identified in rat HCC development in both of protein and mRNA levels (**Figure 2F and G**). Furthermore, we performed immunohistochemistry to examine the expression of FEN1 in a local HCC cohort with 135 self-matched HCC and tissues. As shown in **Figure 2H and I**, samples at advanced TNM stages presented higher expression of FEN1 than para-cancerous tissues and cases at early stages.

### Clinical implications and prognostic significance of FEN1 for HCC

**Figure 3:**
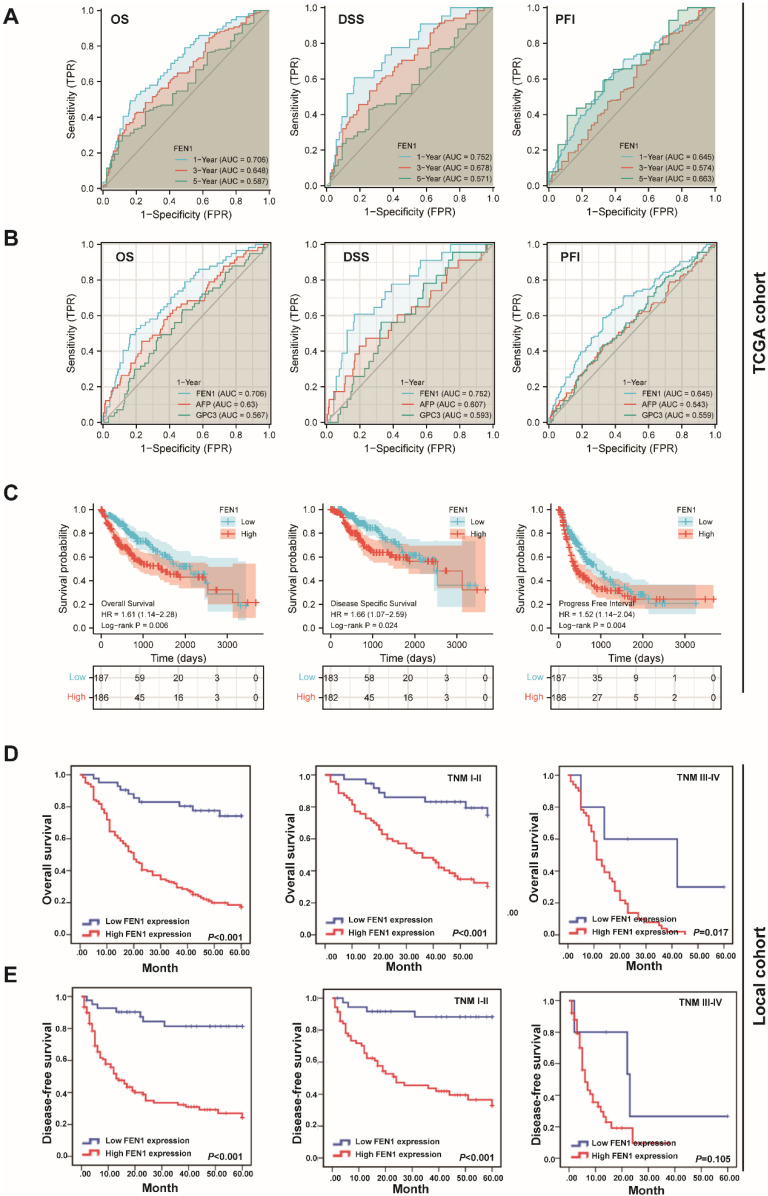
** The prognostic significance of FEN1 for HCC. (A)** Time-dependent ROC of FEN1 for overall survival (OS), disease specific survival (DSS), and progress free interval (PFI) of HCC patients in TCGA cohort. **(B)** The comparison of FEN1 with AFP and GPC3 for OS, DSS, and PFI of HCC patients in TCGA cohort. **(C)** Kaplan-Meier analysis for OS, DSS, and PFI of HCC patients in TCGA cohort. **(D)** Kaplan-Meier analysis for OS of HCC patients in entire local cohort and stratified subgroups.** (E)** Kaplan-Meier analysis for disease free survival (DFS) of HCC patients in entire local cohort and stratified sub-groups.

According to the expression features of FEN1, we further evaluated its clinical significance in HCC. In TCGA cohort, high expression of FEN1 was significantly associated with gender, AFP levels, T stage, pathologic stage, and histologic grade (**Table S2**). Univariate and multivariate Cox regression analyses indicated that pathologic stage and FEN1 expression were independent factors for overall survival (OS) of HCC patients (**Table S3**), while its performance for disease specific survival (DSS) and progress free interval (PFI) was also approximately statistically significant (**Table S4 and Table S5**). As shown in** Figure 3A**, FEN1 exhibited excellent predictive capacity for 1-, 3-, and 5- year of OS, DSS, and PFI according to time-dependent ROC. Remarkably, FEN1 showed higher area under ROC (AUROC) compared with two classical HCC markers (AFP and GPC3; **Figure 3B**). In addition, high-FEN1 patients had obviously shorter OS, DSS, and PFI than FEN1-low patients **(Figure 3C)**. The stratification survival analyses indicated that FEN1 had robustly prognostic capacity for both elder and younger patients, while it showed better performance in patients at early stages **(Figure S2A and B)**. Then, a nomogram consisting of FEN1 expression, pathologic stage, and histologic grade was established to provide accurate prediction of OS, DSS, and PFI of HCC patients, respectively **(Figure S3)**.

Then we further investigated the clinical implications of FEN1 in local HCC cohort. As demonstrated in **Table 1**, FEN1 expression was correlated with HBsAg, multifocal tumors, TNM stage, metastasis, and recurrence. In consistent with the observations in TCGA, univariate and multivariate Cox regression analyses suggested FEN1 expression as an independent factor for the OS and disease-free survival (DFS) in the local HCC cohort **(Table 2 and 3)**. Then, Kaplan-Meier analyses indicated that high expression of FEN1 led to shorter OS and DFS of HCC patients. In addition, high FEN1 expression was associated with shorter OS or DFS of patients at early or advanced TNM stage **(Figure 3D and E)**. Remarkably, FEN1 overexpression also implicated poor OS and DFS of HCC patients administrated with chemotherapy **(Figure S4)**. Thus, FEN1 might be a robustly prognostic marker for HCC patients.

### FEN1 facilitated the aggressive behaviors and EMT process of HCC cells

**Figure 4:**
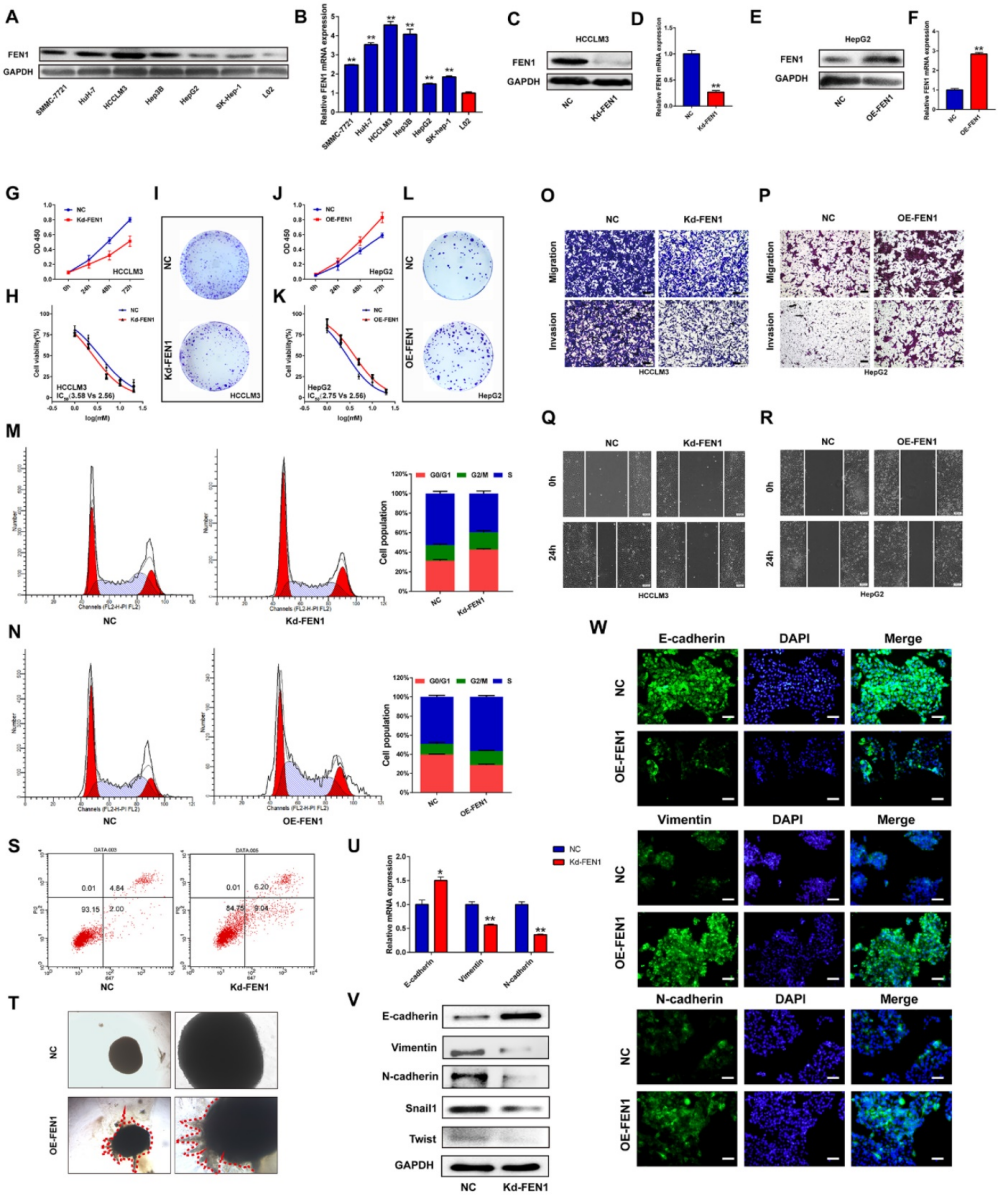
** FEN1 enhanced aggressive behaviors of HCC cells. (A)** Protein levels of FEN1 in HCC cell lines and normal liver cell LO2 were detected by western blotting.** (B)** mRNA levels of FEN1 in HCC cell lines and LO2 were detected by RT-qPCR. **(C, D)** Protein and mRNA levels of FEN1 in HCCLM3 cells transfected with Kd-FEN1 plasmids. **(E, F)** Protein and mRNA levels of FEN1 in HepG2 cells transfected with OE-FEN1 plasmids. **(G-I)** Proliferation, sorafenib sensitivity, and colony formation of HCCLM3 cells transfected with Kd-FEN1 plasmids. **(J-L)** Proliferation, sorafenib sensitivity, and colony formation of HepG2 cells transfected with OE-FEN1 plasmids. (**M, N, S**) Cell cycle distribution and apoptotic ratio were detected by flow cytometry.** (O, P)** The migration and invasion of the HCC cells were detected by transwell assay. **(Q, R)** Wound-healing assays of the HCC cells in different groups. **(T)** 3D invasion assays of HCC cells in NC group and OE-FEN1 group. **(U)** The mRNA expression of EMT markers in HCCLM3 cells transfected with Kd-FEN1 plasmids. **(V)** Protein expression of EMT markers detected by western blotting. **(W)**The immunofluorescence assay for detecting the EMT markers**.** **, P<0.01; *, P<0.05.

Based on the investigations in TCGA and local cohorts, we further discovered the potential functions modulated by FEN1. As shown in** Figure 4A and B**, protein and mRNA levels of FEN1 were significantly elevated in HCC cell lines compared with that in immortalized hepatocyte LO2. Then HCCLM3 and HepG2 were chosen to conduct loss- or gain- of function validations (**Figure 4C-F**). As elucidated in **Figure 4G-I**, knockdown of FEN1 obviously blocked the proliferation, chemoresistance, and colony formation of HCCLM3 cells. In contrast, overexpressing FEN1 enhanced proliferation, colony formation, and chemoresistance of HepG2 cells (**Figure 4J-L**). Moreover, silencing FEN1 led to G0/G1 arrest of HCC cells, while FEN1 overexpression significantly increased the proportion of S phase (**Figure 4M and N**). The elevated apoptotic ratio was also observed in FEN1-silenced HCCLM3 cells (**Figure 4S**). Furthermore, repressing or overexpressing FEN1 remarkably facilitated or attenuated the migration and invasion of HCC cells (**Figure O and P**). FEN1 could also positively modulate the would-healing of HCC cells (**Figure 4Q and R**). Furthermore, FEN1 overexpression robustly enhanced HepG2 cell invasion in a 3D invasion model (**Figure 4T**). Given its effects on invasion and migration, we discovered the regulatory roles of FEN1 in epithelial-mesenchymal transition (EMT). Knockdown of FEN1 significantly downregulated the expression of Vimentin, N-cadherin, Snail1, and Twist, along with the upregulated expression levels of E-cadherin (**Figure 4U and V**). Consistently, the immunofluorescence assay indicated that overexpressing FEN1 lead to the increasing of Vimentin and N-cadherin with decreasing of E-cadherin expression (**Figure 4W**). Therefore, FEN1 might enhance the aggressive phenotypes of HCC cells.

### FEN1 was involved in P53 signaling of HCC cells

**Figure 5:**
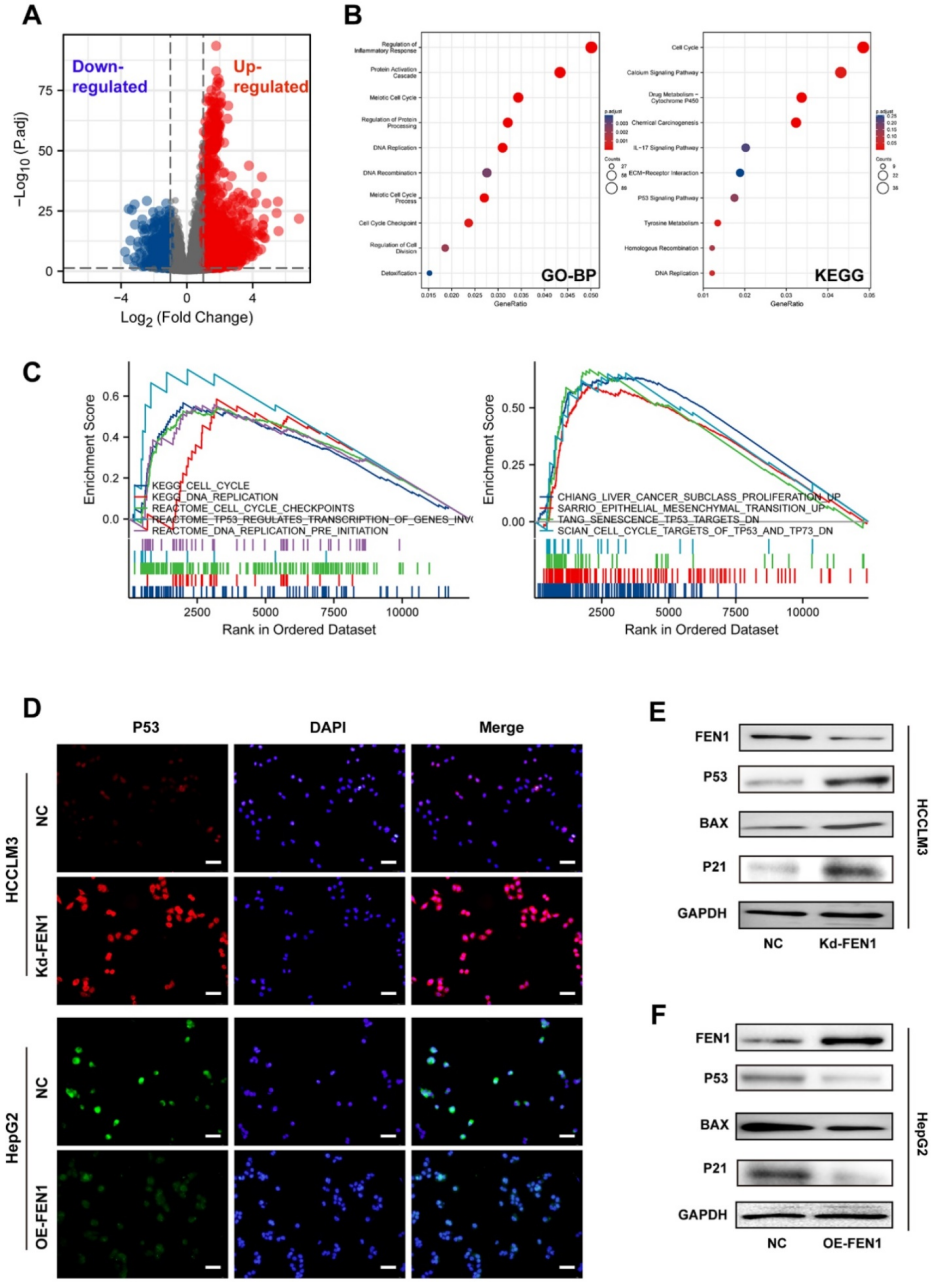
** FEN1 inhibited P53 signaling of HCC cells. (A)** The volcano figure identifying the DEGs between FEN1-high and FEN1-low patients in TCGA dataset.** (B)** KEGG and GO enrichment analyses of the DEGs. **(C)** Gene sets enrichment analysis (GSEA) of the FEN1-mediated pathways in TCGA LIHC cohort. **(D)** The immunofluorescence assays to detect P53 expression with knockdown or overexpressing FEN1. **(E, F)** Protein expression of P53 signaling-related genes in HCC cells. KEGG, Kyoto Encyclopedia of Genes and Genomes; GO_BP, Gene Ontology _ Biological processes.

To further discover the underlying mechanisms, we compared the DEGs between FEN1-high patients and FEN1-low patients in TCGA LIHC cohort** (Figure 5A)**. A total of 3595 DEGs (|log2(FC)|>1 & p.adj<0.05) were identified between the two groups, which were enriched in multiple biological processes, including cell cycle, inflammatory response, protein processing, protein activation cascade, DNA replication, DNA recombination, cell cycle checkpoint, and cell division. DEGs-enriched pathways included cell cycle, Calcium signaling, drug metabolism, IL-17 signaling, P53 signaling, and Tyrosine metabolism** (Figure 5B)**. Consistently, GSEA analysis indicated that FEN1 might participate in various pathways, such as cell cycle, DNA replication, cell cycle checkpoints, TP53-modulated genes transcription, liver cancer proliferation, EMT promotion, senescence TP53 targets, and TP53/TP73 signaling **(Figure 5C)**. Knockdown or overexpression of FEN1 could enhance or decrease the immunofluorescence intensity of P53, respectively (**Figure 5D**). Further molecular validation demonstrated that knockdown of FEN1 significantly upregulated P53, BAX, and P21 expressions in HCCLM3 cells, while overexpression of FEN1 decreased the expression of P53, BAX and P21 in HepG2 cells (**Figure 5E and F**).

### FEN1 inactivated P53 signaling in a USP7/MDM2 dependent manner

**Figure 6:**
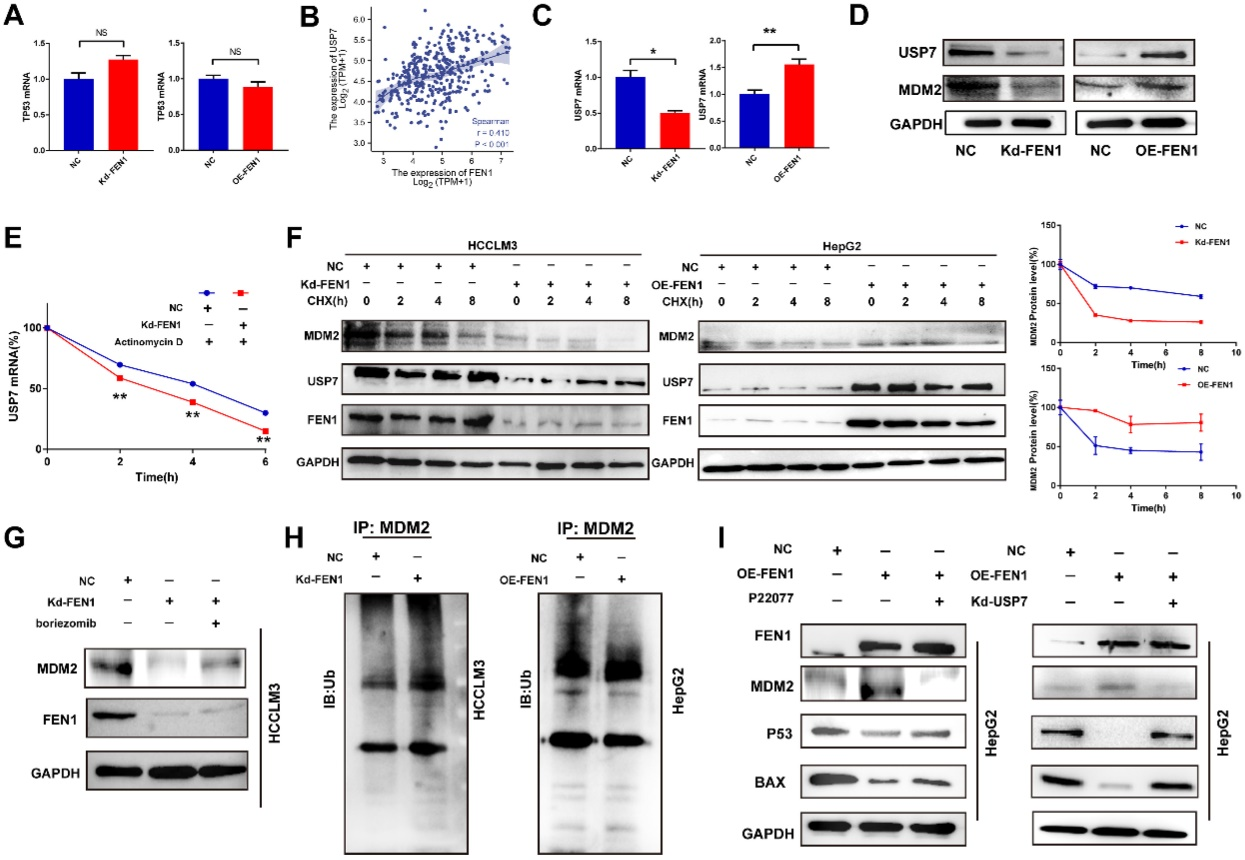
** FEN1 inhibited P53 signaling of HCC cells. (A)** mRNA levels of TP53 in HCCLM3 cells transfected with Kd-FEN1 plasmids and HepG2 cells transfected with OE-FEN1. **(B)** The correlation of FEN1 mRNA with USP7 mRNA in TCGA LIHC data portal. **(C)** mRNA levels of USP7 in HCCLM3 cells transfected with Kd-FEN1 plasmids and HepG2 cells transfected with OE-FEN1 plasmids. (D) Protein levels of USP7 and MDM2 in HCC cells of different groups.** (E)** USP7 mRNA levels of HCC cells in different groups administrated with actinomycin D.** (F)** Expression of MDM2 in HCCLM3 cells or HepG2 cells treated with CHX (100 μM) for the indicated times.** (G)** The expression of MDM2 protein expression in FEN1-silenced HCCLM3 cells treated with boriezomib. **(H)** Ubiquitination of MDM2 in FEN1-silenced HCCLM3 cells and FEN1-overexpressed HepG2 cells. **(I)** The protein levels of MDM2/P53 signaling in FEN1-overexpressed HepG2 cells with USP7 inhibitor P22077 or siRNA. **, P<0.01; *, P<0.05.

P53 acts as a crucial tumor-suppressive gene in multiple malignancies. Then we explored the potential mechanism that FEN1 regulated P53 expression. However, knockdown or overexpression of FEN1 brought no significant alterations in P53 at mRNA levels **(Figure 6A)**. Thus, we speculated that FEN1 might modulate P53 through post-transcriptional modifications. Previous studies indicated that USP7/MDM2 was an important post-transcriptional regulatory axis for P53, suggesting USP7 was an important regulator for P53 activity. In addition, P22077 could significantly inhibit the aggressive features of HCC cells, including proliferation, migration and invasion **(Figure S5)**. Based on the TCGA LIHC data portal, FEN1 mRNA level was significantly correlated with USP7 expression **(Figure 6B)**.

Knockdown or overexpression of FEN1 downregulated or elevated the expression of USP7 at mRNA levels, respectively **(Figure 6C)**. MDM2 was a subtract of USP7 and an E3 ubiquitin-protein ligase for P53. Knockdown or overexpression of FEN1 decreased or increased the protein expression of USP7 and MDM2, respectively **(Figure 6D)**. In addition, FEN1 silencing accelerated the degradation of USP7 induced by Actinomycin D, suggesting the potential roles of FEN1 in USP7 mRNA stabilization **(Figure 6E)**. Furthermore, repressing FEN1 significantly accelerated the degradation of MDM2 induced by CHX, while exogenous FEN1 attenuated the degradation of MDM2 (**Figure 6F**). Consistently, knockdown of FEN1 significantly decreased MDM2 expression of HCCLM3 cells, which was further rescued by protease inhibitor boriezomib (**Figure 6G**). Subsequently, we further investigated the FEN1/USP7 mediated de-ubiquitination effects on MDM2 protein. As expected, the enhanced MDM2 ubiquitination from the MDM2-lysates immunocomplex was observed in FEN1-silenced HCCLM3 cells, while the decreased ubiquitination level of MDM2 was detected in FEN1-overexpressed HepG2 cells **(Figure 6H)**. Subsequently, a specific inhibitor of USP7 (P22077) or knockdown of USP7 rescued the alterations of MDM2 /P53 signaling induced by FEN1 overexpression **(Figure 6I)**. Therefore, FEN1 might inactivate P53 signaling through USP7-mediated de-ubiquitination on MDM2.

### USP7 inhibitor reversed the aggressive behaviors induced by FEN1

**Figure 7:**
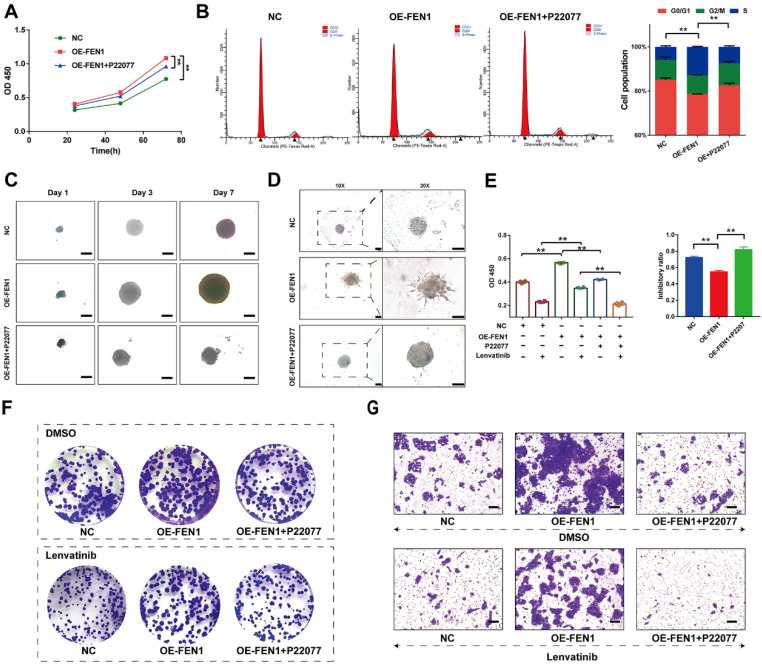
** FEN1 enhanced the malignant behaviors in a USP7-dependent manner. (A)** The proliferation of HepG2 cells of indicating groups was detected by CCK-8 assay.** (B)** Cell cycle of HCC cells was detected by flowcytometry.** (C)** The growth of the 3D spheroids derived from HepG2 cells in each group on 1^st^, 3^rd^, and 7^th^ Day. **(D)** 3D invasion assays in spheroids derived from HepG2 cells.** (E)** The proliferation of HepG2 cells treated with Lenvatinib of indicating groups was detected by CCK-8 assay. **(F)** The colony formation of each groups following Lenvatinib treatment.** (G)** The migration of cells in each group was detected by transwell assay. **, P<0.01; *, P<0.05.

We further investigated the effects of FEN1 suppressing on FEN1-mediated malignant phenotypes of HCC cells. As shown in** Figure 7A**, P22077 significantly abrogated the FEN1 overexpression-induced proliferation of HepG2 cells. In addition, FEN1 overexpression obviously decreased the ration of G0/G1 phase, while P22077 reversed the effects of exogenous FEN1 expression on cell cycle of HCC cells **(Figure 7B)**. Next, 3D spheroid model to further investigate the function of FEN1/USP7 axis in tumor growth and invasion. Ectopic expression of FEN1 accelerated the growth and invasion of the 3D spheroids derived from HepG2 cells. In contrast, administration of P22077 impeded the growth and invasion of the spheroids** (Figure 7C&D)**. Additionally, blockage of USP7 by P22077 also improved the Lenvatinib-induced inhibition of proliferation in FEN1-overexpressed HepG2 cells **(Figure 7E)**. As elucidated in **Figure 7F&G**, co-administration of P22077 and Lenvatinib robustly abrogate the enhancement of aggressive phenotypes induced by FEN1 overexpression. The results above indicated that FEN1 might promote the malignant behaviors in a USP7-dependent manner.

### FEN1 facilitated tumor growth *in vivo*

**Figure 8:**
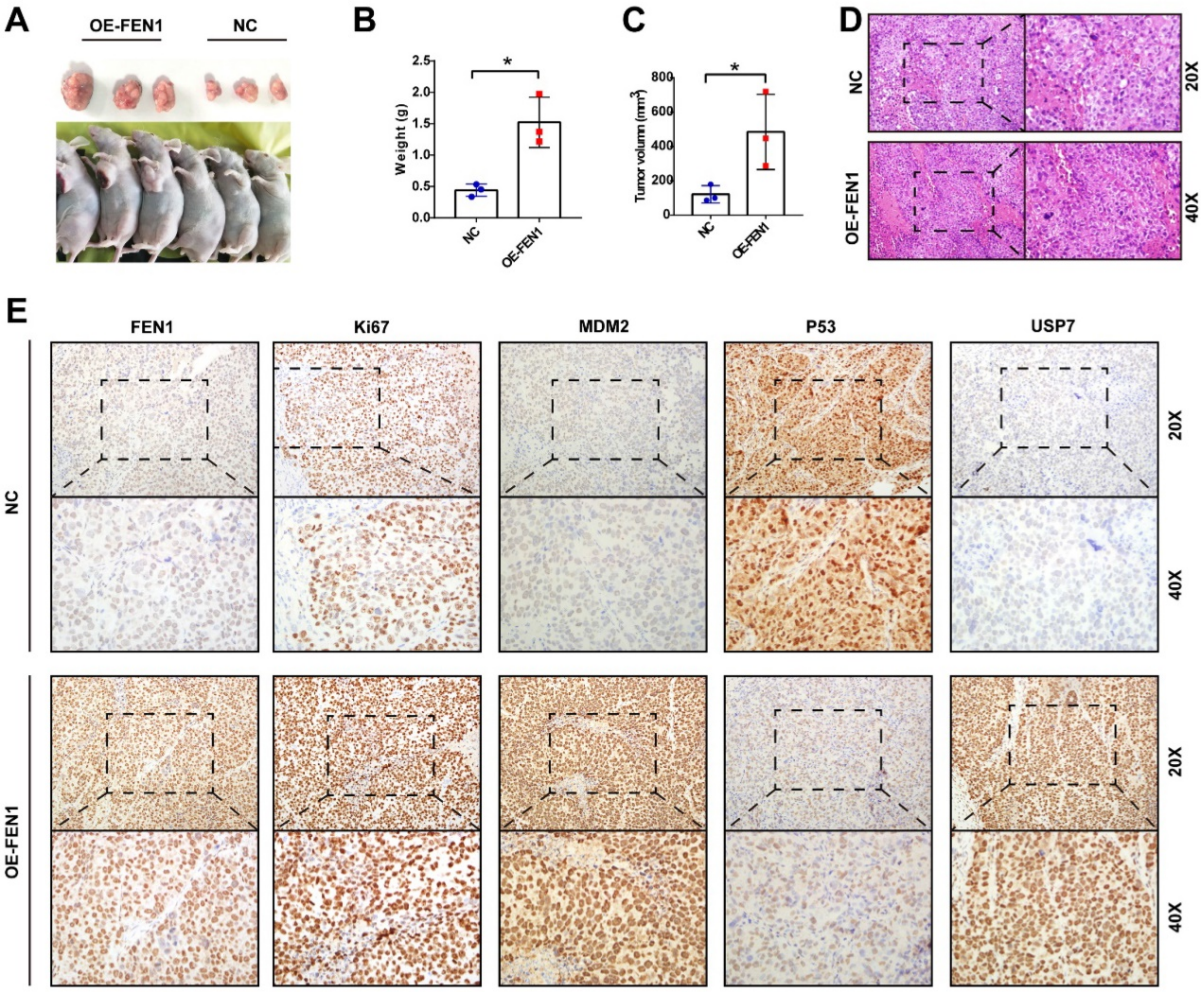
** FEN1 promoted HCC growth* in vivo*. (A)** The HepG2-derived xenograft tumors of NC group and OE-FEN1 groups. **(B, C)** The volume and weight of HepG2-derived xenograft tumors in NC group and OE-FEN1 groups. **(D)** The representative H&E staining of the xenograft tumors in NC group and OE-FEN1 groups. **(E)** The representative immunohistochemical staining (FEN1, P53, USP7, Ki67, and MDM2) of the xenograft tumors. *, P<0.05.

Ultimately, we validated the oncogenic roles of FEN1 in the xenograft model. As shown in** Figure 8A-C,** FEN1-overexpressed HepG2-derived xenograft tumors had larger volume and heavier weight than these of NC group. In addition, the H&E staining showed that FEN1 overexpression might cause typically histological changes in the xenografts **(Figure 8D).** Furthermore, immunohistochemical staining demonstrated that FEN1 overexpression significantly elevated the expression of Ki67, MDM2, and USP7 of the xenograft tumor tissues, while the exogenous FEN1 remarkably decreased P53 expression **(Figure 8E)**.

## Discussion

HCC is a dynamic and multi-central process with activation of various oncogenes and inactivation of tumor suppressors 21. Upon multi-omics analyses, numerous studies try to find the vital genes during hepatocarcinogenesis and HCC progression, which could robustly provide prognostic biomarkers or potential therapeutic targets 22. In the current study, we included 4 GEO datasets to identify the key co-overexpressed genes in HCC tissues. These genes were mainly enriched in cell cycle, metabolism, inflammatory pathways, suggesting the potential roles of the genes in HCC occurrence and progression. FEN1, with top node degree in interaction network and enriched frequency, was selected for further investigation.

Previous studies have indicated that FEN1 was overexpressed in multiple solid tumors like lung cancer and breast cancer with aggressive clinical implications 11, 12. In addition to its significance, in situ sensing of oncogenic FEN1 with DNA nanosphere or dumbbell DNA probe has been developed for early diagnosis and precision medicine 23, 24. Actually, a recent study noted that FEN1 expression was elevated in HCC tissues by analyzing bioinformatic data 25. Consistently, in our current study, overexpression of FEN1 was also identified in HCC tissues and advanced HCC cases based on multiple bioinformatic datasets and local HCC cohort. Additionally, HCC tissues had significantly higher FEN1 levels than HCC precursor status or liver cirrhosis tissues, suggesting the oncogenic role of FEN1 in HCC. Then, we examined the FEN1 expression in different stages of hepatocarcinogenesis in a pre-established rat model. Dynamically increased expression of FEN1 was observed from degeneration, pre-cancerous status to HCC, indicating that FEN1 might participate in the occurrence of HCC. Moreover, aberrant expression of FEN1 was correlated with higher AFP levels, advanced stages/ grades, and metastasis in bioinformatic and local HCC cohorts. Remarkably, overexpression of FEN1 might cause shorter OS, DSS, and PFI. The Cox regression analyses identified FEN1 as an independent predictor for the survival of HCC patients. Thus, the observations in clinical samples indicated that FEN1 was a potential biomarker for the prognosis and progression of HCC. Given the expression features and clinical implications, we further evaluated its effects on the biological functions of HCC cells. Silencing or overexpressing FEN1 could significantly inhibit or facilitate the aggressive phenotypes of HCC cells, including proliferation, chemoresistance, migration, invasion, and EMT process. It suggested that FEN1 might also be a promising molecular target for HCC.

It is reasonable that FEN1 might enhance the malignant behaviors through activate or inactivate tumor-promotors or tumor-suppressors. P53 is a canonical tumor-suppressor that regulates various tumor-related processes, such as proliferation, migration, chemosensitivity, senescence, and apoptosis 26. P53 mutation and deficiency occur most frequently in HCC patients, abrogating its tumor-suppressor activity to facilitate hepatocarcinogenesis 27. Thus, recovering the activity of P53 signaling it is a promising strategy for the treatment of HCC patients. Attractively, based on the GSEA upon the DEGs between FEN1 high- and FEN1 low- HCC cases, it was speculated that FEN1 might modulate the activity of P53 signaling. Further validations found that overexpression of FEN1 inactivated P53 signaling, while knockdown of FEN1 recovered the activity of P53 signaling. Then we tried to find the regulatory mechanisms of FEN1 on P53. Firstly, we aimed to ensure if it belonged to transcriptional regulation. However, FEN1 induced no obvious changes in TP53 mRNA levels. Therefore, we speculated that it might be a post-transcriptional regulation.

Numerous studies have demonstrated that MDM2 is a nuclear-localized E3 ubiquitin ligase response for proteasomal degradation of P53 28, 29. Additionally, MDM2 has been reported as a substrate of USP7, which was a liver oncogenic DUB 30, 31. Thus, we further validated whether FEN1 regulated P53 through USP7/MDM2 axis. As expected, FEN1 could enhance USP7 expression at both of the mRNA and protein levels. Moreover, FEN1 silencing accelerated the degradation of USP7 induced by Actinomycin D, suggesting that FEN1 might upregulate USP7 expression by stabilizing its mRNA. Furthermore, exogenous FEN1 obviously alleviated the degradation of MDM2 induced by CHX. Protease inhibitor boriezomib rescued the MDM2 expression downregulated by FEN1 silencing, indicating that FEN1 could modulate its degradation by protease. Therefore, we further discovered its effects on ubiquitin modifications. Ubiquitin binding assay demonstrated that that FEN1 could remove ubiquitination of MDM2, while knockdown of USP7 with P22077 further abrogated the upregulation of MDM2 induced by FEN1. It suggested that FEN1 might recruit USP7 to stabilize MDM2 protein, subsequently promoting the degradation of P53. Additionally, P22077 also rescued the effects of ectopic FEN1 expression on aggressive phenotypes, including proliferation, colony formation, migration, invasion, and Lenvatinib resistance. In addition to the typically malignant behaviors, the phenotype rescue assay further implied that FEN1 might be potential targets to overcome the resistance to targeted agents. P53 Loss-function would induce resistance of chemotherapy and targeted therapy, which has been frequently highlighted. Thus, it was proposed that FEN1 might render Lenvatinib resistance to HCC cells by promoting USP7/MDM2 axis-mediated P53 destabilization. It suggested that FEN1 might accelerate HCC progression in a USP7-dependent manner. In consistence, the regulatory cascade was further validated in the xenograft tumors derived from HCC cells with FEN1 overexpression. Though the current results are promising, the study has some limitations. The size of local cohort is relatively small, and large multi-central cohorts are needed to evaluate the clinical significance of FEN1. In addition, FEN1/USP7/MDM2 axis proposed in this study showed targeted value for HCC treatment, while the deeper regulatory mechanisms should be further investigated by more molecular assays and strategies.

## Conclusion

The current study identified that the hub gene FEN1 overexpression facilitated the malignant behaviors of HCC cells by inactivating P53 signaling via enhancing USP7/MDM2 axis, suggesting that FEN1 might serve as a therapeutic target and prognostic marker for HCC. Further explorations are warranted to verify the exact prognostic value in larger cohorts and unveiling underlying mechanisms.

## Supplementary Material

Supplementary figures and tables.

## Figures and Tables

**Table 1:** Clinicopathological features of FEN1 expression in 163 HCC tissues

Group	n	Pos. n (%)	*χ*^2^ value	*P* value	
Age					
≤ 60	85	62(72.94)	0.155		0.694
>60	78	59(75.64)			
Gender					
Female	20	12(60.00)	2.414		0.120
Male	143	109(76.22)			
AFP (ng /mL)					
≤ 50	63	44(69.84)	1.036		0.309
> 50	100	77(77.00)			
HBsAg					
Negative	24	13(54.16)	5.925		0.015
Positive	139	108(77.70)			
Tumor size					
≤ 5 cm	89	61(68.54)	0.068		3.323
>5 cm	74	60(81.08)			
Liver cirrhosis					
Without	39	29(74.36)	0.000		0.984
With	124	92(74.19)			
Differentiation degree					
Well	45	34(75.56)	0.057		0.812
Moderate &Poor	118	87(73.73)			
Portal vein invasion					
Without	65	48(73.87)	0.008		0.927
With	98	73(74.49)			
Gross classification					
Unifocal	133	93(69.92)	7.013		0.008
Multifocal	30	28(93.33)			
TNM					
I&II	107	70(65.42)	12.645		<0.001
III&IV	56	51(91.07)			
Metastasis					
Without	132	91(68.94)	10.169		0.001
With	31	30(96.77)			
Recurrence					
No	82	47(57.32)	24.686		<0.001
Yes	81	74(9.36)			
					

**FEN1**, Flap endonuclease 1; **Pos. n (%)**, positive number (%). **TNM**, Tumor node metastasis; **AFP,** alpha- fetoprotein.

**Table 2:** Univariate and multivariate analysis for identifying the risk factors of overall survival in HCC patients

Group	Univariate			Multivariate (gender and sex adjusted)		
	HR	*P*	95% CI	HR	*P*	95% CI
Gender						
Male *vs.* Female	0.715	0.087	0.487-1.050			
Age (years)						
≤60 *vs.*>60	1.399	0.312	0.730-2.638			
Tumor diameter (cm)						
≤5 *vs.*>5	3.118	<0.001	2.098-4.634	2.037	0.001	1.315-3.154
Differentiation						
Well *vs.* Moderate & poor	1.811	0.011	1.148-2.857	1.872	<0.001	1.338-2.617
AFP (ng/mL)						
≤50 *vs.*>50	1.799	0.005	1.199-2.699	1.284	0.258	0.832-1.982
Liver cirrhosis						
Yes *vs.* No	0.988	0.956	0.645-1.514			
Gross classification						
Multifocal *vs.* unifocal	2.354	<0.001	1.510-3.668	1.751	0.021	1.088-2.819
Portal vein invasion						
Yes *vs.* no	1.806	0.004	1.206-2.705	1.457	0.089	0.945-2.246
HBsAg						
Yes *vs.* No	2.462	0.007	1.282-4.729	1.236	0.552	0.615-2.486
TNM						
I-II *vs.* III-IV	4.597	<0.001	3.058-6.911	2.724	<0.001	0.618-1.664
Metastasis						
Yes *vs.* No	2.848	<0.001	1.831-4.432	1.014	0.955	1.827-6.737
FEN1 expression						
High *vs.* low	5.668	<0.001	2.943-10.916	4.905	<0.001	2.453-9.808

**FEN1**, Flap endonuclease 1; **TNM**, tumor node metastasis; **AFP**, alpha-fetoprotein.

**Table 3:** Univariate and multivariate analysis for identifying the risk factors of disease-free survival in HCC patients

Group	Univariate				Multivariate (gender and sex adjusted)		
	HR	*P*		95% CI	HR	*P*	95% CI
Gender							
Male *vs.* Female	0.878	0.559		0.567-1.359			
Age (years)							
≤60 *vs.*>60	1.901	0.130		0.827-4.369			
Tumor diameter (cm)							
≤5 *vs.*>5	1.690	0.021		1.083-2.637	0.998	0.993	0.608-1.637
Differentiation							
Well *vs.* Moderate & poor	1.013	0.958		0.631-1.625			
AFP (ng/mL)							
≤50 *vs.*>50	1.498	0.083		0.949-2.363			
Liver cirrhosis							
Yes *vs.* No	0.904	0.696	0.545-1.499				
Gross classification							
Multifocal *vs.* unifocal	2.648	<0.001		1.629-4.306	2.062	0.004	1.254-3.391
Portal vein invasion							
Yes *vs.* no	1.353	0.188		0.862-2.122			
HBsAg							
Yes *vs.* No	2.038	0.057		0.980-4.236			
TNM							
I-II *vs.* III-IV	3.517	<0.001		2.205-5.609	2.814	<0.001	1.658-4.776
Metastasis							
Yes *vs.* No	1.213	0.527		0.667-2.208			
FEN1 expression							
High *vs.* low	6.455	<0.001		2.957-14.091	4.813	<0.001	2.150-10.773
							

**FEN1**, Flap endonuclease 1;** TNM**, Tumor node metastasis; **AFP**, alpha- fetoprotein.

## Abbreviations

HCC: Hepatocellular carcinoma
HBV: hepatitis B virus
HCV: hepatitis C virus
FEN1: Flap endonuclease 1
DEGs: differential expression genes
EMT: epithelial-mesenchymal transition
MDM2: mouse double minute 2
USP7: ubiquitin-specific protease 7
GEN: gap endonuclease
cccDNA: covalently closed circular DNA
TPM: transcripts per million
FPKM: Fragments Per Kilobase per Million
FDR: False discovery rate
NCBI: National Center for Biotechnology Information
GEO: Gene Expression Omnibus
TCGA-LIHC: The Cancer Genome Atlas Liver Hepatocellular Carcinoma
GSEA: Gene set enrichment analysis
CCK-8: Cell Counting Kit-8 assay
PFA: paraformaldehyde
RIPA: radioimmunoprecipitation assay buffer
PI: Propidium iodide
SDS: sodium dodecyl sulfate
PVDF: polyvinylidene difluoride
HPR: horseradish peroxidase
ECL: enhanced chemiluminescence
GAPDH: glyceraldehyde phosphate dehydrogenase
GO: gene ontology
OS: overall survival
DFS: disease free survival
DSS: disease specific survival
PFI: progress free interval
