# Supplementary material for: Flap endonuclease 1 Facilitated Hepatocellular Carcinoma Progression by Enhancing USP7/MDM2-mediated P53 Inactivation
Source: Int J Biol Sci. 2022 Jan 1;18(3):1022–38. doi: 10.7150/ijbs.68179 (PMC8771828; doi:10.7150/ijbs.68179)
Supplement: Supplementary file 1 — Supplementary figures and tables. [file ijbsv18p1022s1.pdf]

Supplemental Tables

Table S1. The primers of RT-qPCR.

Gene	Species	Sequences (5'-3')
FEN1	Human	F: ATGACATCAAGAGCTACTTTGGC
		R: GGCGAACAGCAATCAGGAACT
USP7	Human	F: GATGATTCGTCTAAAAGCGTCC
		R: GAATAATTTGGGTATGGTGCCC
TP53	Human	F: TTCCTGAAAACAACGTTCTGTC
		R: AACCATTGTTCAATATCGTCCG
GAPDH	Human	F: AGGTCGGTGTGAACGGATTTG
		R: GGGGTCGTTGATGGCAACA
VIM	Human	F: GACGCCATCAACACCGAGTT
		R: CTTTGTCGTTGGTTAGCTGGT
CDH1	Human	F: CGAGAGCTACACGTTCACGG
		R: GGGTGTCGAGGGAAAAATAGG
CDH2	Human	F: TCAGGCGTCTGTAGAGGCTT
		R: ATGCACATCCTTCGATAAGACTG
GAPDH	Mouse	F: AGGTCGGTGTGAACGGATTTG
		R: TGTAGACCATGTAGTTGAGGTCA
FEN1	Mouse	F: TTCACGGCCTTGCCAAACTAA
		R: ACAGCAATCAGGAACTGGTAGA

Table S2. The correlation of FEN1 expression with clinicopathological features in TCGA LIHC cohort.

Characteristics	Total(N)	Odds Ratio(OR)	P value
Gender (Male vs. Female)	374	0.567 (0.364-0.878)	**0.011**
Age (>60 vs. <=60)	373	0.686 (0.455-1.031)	0.070
AFP(ng/ml) (>400 vs. <=400)	280	2.516 (1.422-4.557)	**0.002**
T stage (T3&T4 vs. T1&T2)	371	1.624 (1.012-2.627)	**0.046**
N stage (N1 vs. N0)	258	2.687 (0.339-54.709)	0.395
M stage (M1 vs. M0)	272	0.914 (0.108-7.710)	0.929
Pathologic stage (Stage III&Stage IV vs. Stage I&Stage II)	350	1.697 (1.046-2.777)	**0.033**
Histologic grade (G3&G4 vs. G1&G2)	369	2.751 (1.780-4.293)	**<0.001**
Child-Pugh grade (B&C vs. A)	241	0.488 (0.180-1.206)	0.133
Vascular invasion (Yes vs. No)	318	1.234 (0.777-1.962)	0.374
BMI (>25 vs. <=25)	337	0.690 (0.448-1.060)	0.091
Residual tumor (R1&R2 vs. R0)	345	1.044 (0.398-2.738)	0.930
Adjacent hepatic tissue inflammation (Mild& Severe vs. None)	237	0.889 (0.532-1.483)	0.652
Albumin(g/dl) (>=3.5 vs. <3.5)	300	1.076 (0.628-1.855)	0.790
Prothrombin time (>4 vs. <=4)	297	0.654 (0.393-1.078)	0.098
Fibrosis ishak score (3/4 & 5/6 vs. 0 & 1/2)	215	1.283 (0.750-2.202)	0.364

FEN1, Flap endonuclease 1; **AFP,** alpha- fetoprotein; **BMI**, Body Mass Index. **Bold,** $P<0.05$.

Table S3. Univariate and multivariate analysis for identifying the risk factors of overall survival in TCGA LIHC cohort.

Characteristics	Total(N)	Univariate analysis		Multivariate analysis	
		Hazard ratio (95% CI)	P value	Hazard ratio (95% CI)	P value
Gender (Male vs. Female)	373	0.793 (0.557-1.130)	0.200		
Age (>60 vs. <=60)	373	1.205 (0.850-1.708)	0.295		
Pathologic stage (Stage III&Stage IV vs. Stage I&Stage II)	349	2.504 (1.727-3.631)	**<0.001**	2.320 (1.590-3.385)	**<0.001**
Histologic grade (G3&G4 vs. G1&G2)	368	1.091 (0.761-1.564)	0.636		
Adjacent hepatic tissue inflammation (Mild&Severe vs. None)	236	1.194 (0.734-1.942)	0.475		
AFP(ng/ml) (>400 vs. <=400)	279	1.075 (0.658-1.759)	0.772		
Child-Pugh grade (B&C vs. A)	240	1.643 (0.811-3.330)	0.168		
Vascular invasion (Yes vs. No)	317	1.344 (0.887-2.035)	0.163		
Fibrosis ishak score (3/4&5/6 vs. 0&1/2)	214	0.740 (0.445-1.232)	0.247		
FEN1 (High vs. Low)	373	1.617 (1.141-2.292)	**0.007**	1.495 (1.023-2.184)	**0.038**

FEN1, Flap endonuclease 1; **AFP,** alpha- fetoprotein. **Bold,** *P*<0.05.

Table S4. Univariate and multivariate analysis for identifying the risk factors of Disease specific survival in TCGA LIHC cohort.

Characteristics	Total(N)	Univariate analysis		Multivariate analysis	
		Hazard ratio (95% CI)	P value	Hazard ratio (95% CI)	P value
Gender (Male vs. Female)	365	0.813 (0.516-1.281)	0.373		
Age (>60 vs. <=60)	365	0.846 (0.543-1.317)	0.458		
Pathologic stage (Stage III&Stage IV vs. Stage I&Stage II)	341	3.803 (2.342-6.176)	**<0.001**	2.652 (1.345-5.231)	**0.005**
Histologic grade (G3&G4 vs. G1&G2)	360	1.086 (0.683-1.728)	0.726		
Adjacent hepatic tissue inflammation (Mild&Severe vs. None)	232	1.403 (0.768-2.566)	0.271		
AFP(ng/ml) (>400 vs. <=400)	275	0.867 (0.450-1.668)	0.668		
Child-Pugh grade (B&C vs. A)	235	2.560 (1.123-5.834)	**0.025**	2.993 (1.254-7.142)	**0.014**
Vascular invasion (Yes vs. No)	309	1.277 (0.707-2.306)	0.418		
Fibrosis ishak score (3/4&5/6 vs. 0&1/2)	210	0.660 (0.340-1.279)	0.218		
FEN1 (High vs. Low)	365	1.668 (1.065-2.611)	**0.025**	1.967 (0.986-3.925)	0.055

FEN1, Flap endonuclease 1; **AFP,** alpha- fetoprotein. **Bold,** *P*<0.05.

Table S5. Univariate and multivariate analysis for identifying the risk factors of Progress free interval in TCGA LIHC cohort.

Characteristics	Total(N)	Univariate analysis		Multivariate analysis	
		Hazard ratio (95% CI)	P value	Hazard ratio (95% CI)	P value
Gender (Male vs. Female)	373	0.982 (0.721-1.338)	0.909		
Age (>60 vs. <=60)	373	0.960 (0.718-1.284)	0.783		
Pathologic stage (Stage III&Stage IV vs. Stage I&Stage II)	349	2.201 (1.591-3.046)	**<0.001**	1.726 (1.146-2.600)	**0.009**
Histologic grade (G3&G4 vs. G1&G2)	368	1.152 (0.853-1.557)	0.355		
Adjacent hepatic tissue inflammation (Mild&Severe vs. None)	236	1.238 (0.867-1.768)	0.241		
AFP(ng/ml) (>400 vs. <=400)	279	1.045 (0.698-1.563)	0.832		
Child-Pugh grade (B&C vs. A)	240	1.395 (0.765-2.545)	0.277		
Vascular invasion (Yes vs. No)	317	1.676 (1.196-2.348)	**0.003**	1.420 (0.985-2.046)	0.060
Fibrosis ishak score (3/4&5/6 vs. 0&1/2)	214	1.209 (0.835-1.750)	0.315		
FEN1 (High vs. Low)	373	1.526 (1.141-2.041)	**0.004**	1.386 (0.980-1.960)	0.065

FEN1, Flap endonuclease 1; **AFP,** alpha- fetoprotein. **Bold,** *P*<0.05.

Supplemental Figures

Dataset	P-value	Type	Nums	Mean	STD	IQR
**GSE22058**	4.540e-32	HCC	100	10.46	0.7488	1.252
		Adjacent	97	9.157	0.4605	0.5743
**GSE25097**	4.340e-50	HCC	268	2.578	1.443	1.955
		Adjacent	243	0.9290	0.3742	0.3715
		Cirrhotic	40	1.063	0.4145	0.4202
		Healthy	6	1.033	0.4268	0.5775
**GSE36376**	4.260e-83	HCC	240	8.054	0.4655	0.7020
		Adjacent	193	7.185	0.1777	0.2225
**GSE14520**	2.740e-77	HCC	225	5.867	0.7973	1.164
		Adjacent	220	4.395	0.3438	0.3245
**GSE10143**	0.9446	HCC	80	10.17	0.6593	0.5700
		Adjacent	82	10.17	0.7600	0.5225
**GSE46444**	0.003339	HCC	88	7.997	0.7973	1.406
		Adjacent	48	8.447	0.8492	1.104
**GSE54236**	1.740e-7	HCC	81	8.011	1.031	1.309
		Adjacent	80	7.280	0.5904	0.5890
**GSE63698**	1.200e-32	HCC	228	6.295	0.8768	1.201
		Adjacent	168	5.408	0.4348	0.5678
**TCGA-LIHC**	7.950e-47	HCC	351	9.587	0.8543	1.265
		Adjacent	49	8.063	0.3662	0.3200
**GSE64041**	2.910e-10	HCC	60	7.233	0.4478	0.5279
		Adjacent	60	6.770	0.2337	0.2727
**GSE76427**	7.430e-18	HCC	115	8.879	0.6040	0.6675
		Adjacent	52	8.190	0.3110	0.2200
**ICGC-LIRI-JP**	1.910e-61	HCC	212	3.135	0.8414	1.235
		Adjacent	177	1.771	0.4024	0.4700

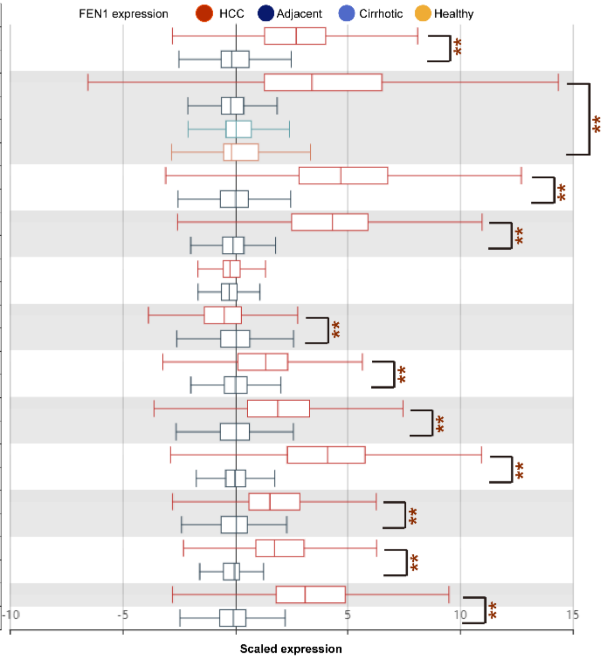

Figure S1. FEN1 expression in multiple bioinformatic datasets.

The FEN1 expression of HCC tissues and adjacent tissues derived from multiple GEO datasets, TCGA, and ICGC datasets.

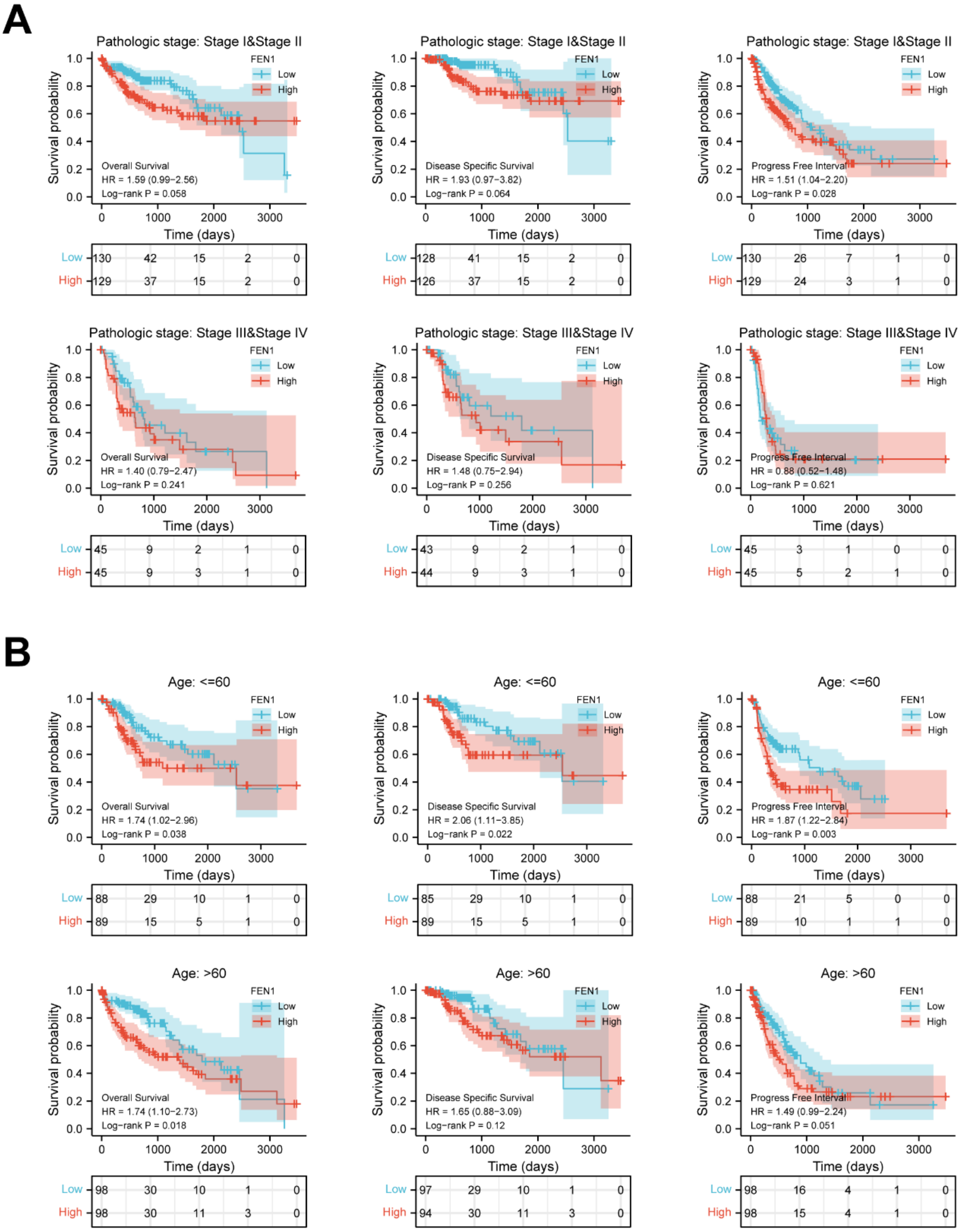

Figure S2. Stratification analysis of the survival in HCC patients in TCGA data portal. (A) The Kaplan-Meier analysis in HCC patients with different pathologic stages. **(B)** The Kaplan-Meier analysis in HCC patients with different age status.

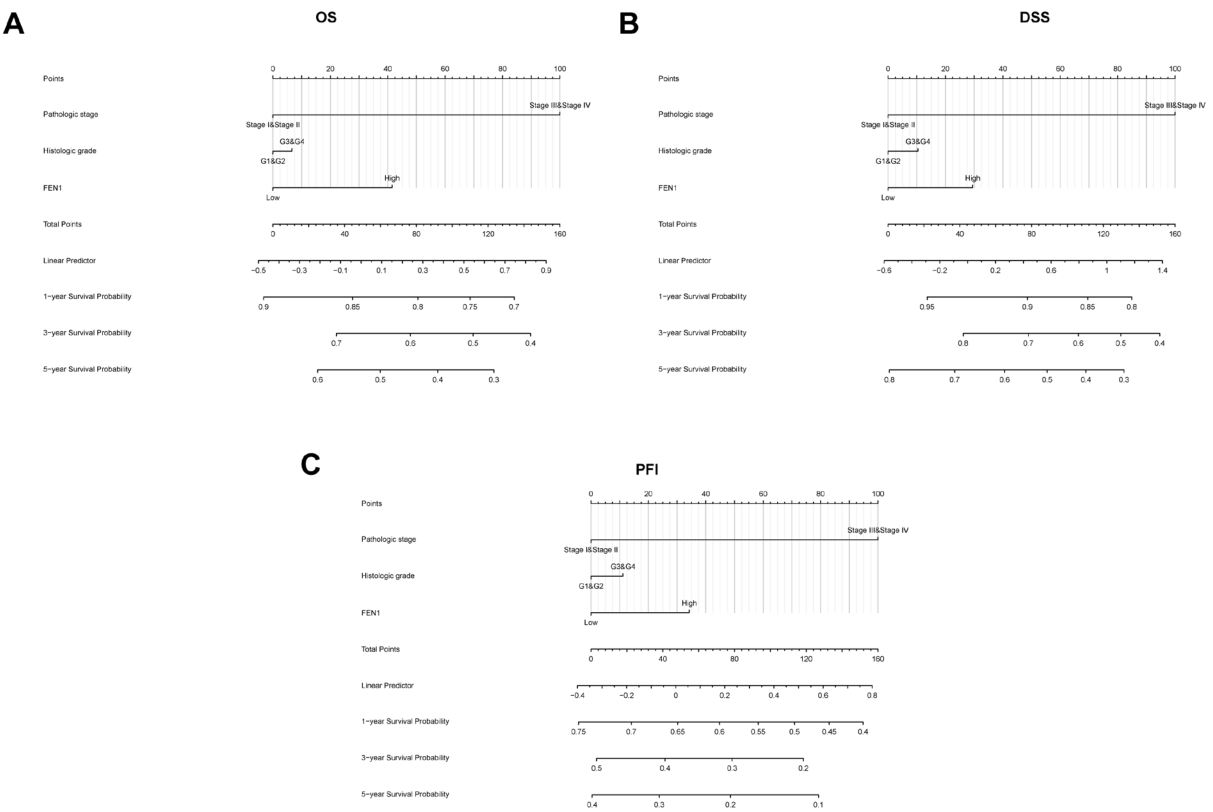

Figure S3. Construction of a FEN1-based nomogram for HCC patients.

(A) The nomogram for OS of HCC patients in TCGA LIHC dataset. **(B)** The nomogram for DSS of HCC patients in TCGA LIHC dataset. **(C)** The nomogram for PFI of HCC patients in TCGA LIHC dataset.

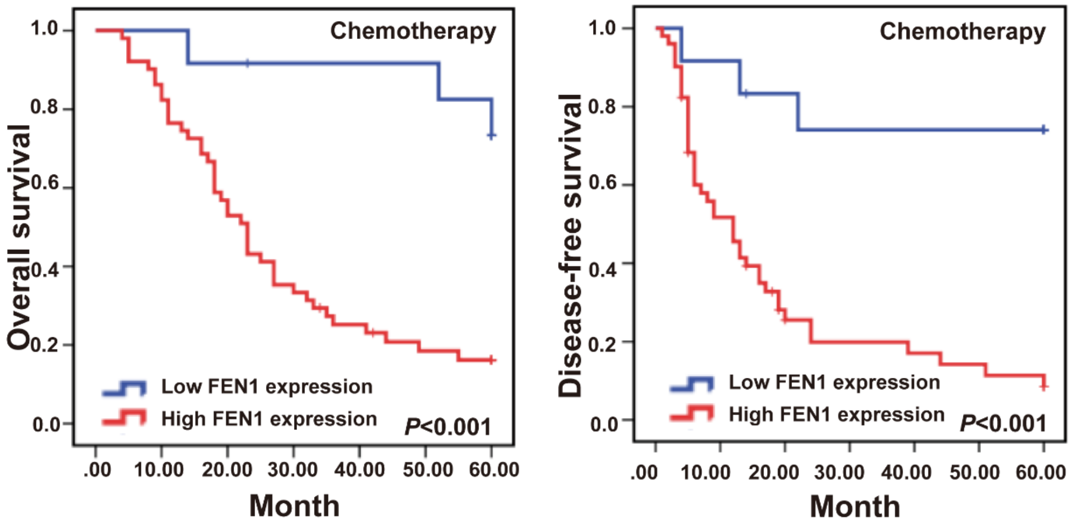

Figure S4. The prognostic significance of FEN1 in HCC patients with chemotherapy. The Kaplan-Meier analysis in FEN1-high or FEN1-low HCC patients treated with chemotherapy.

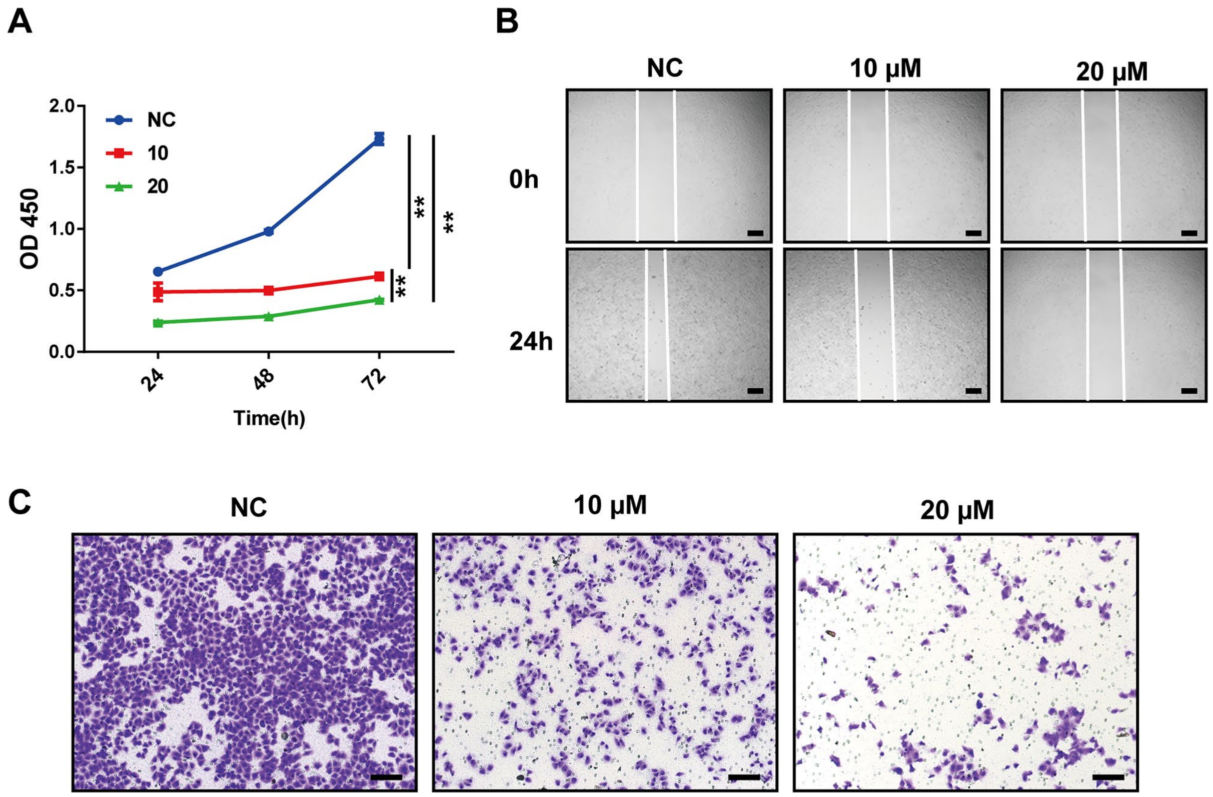

Figure S5. The effects of P22077 on HCC cells. (A) The proliferation of HCCLM3 cells was detected by CCK8 assay following P22077 treatment at different doses. **(B)** The migration of HCCLM3 cells was detected by wound-healing assay. **(C)** The effects of P22077 on the invasion of HCCLM3 cells was detected by transwell assay.
